# Circular economy approaches to microbially-induced carbonate precipitation for bioprocessing of geothermal brine for lithium recovery

**DOI:** 10.1039/d5ra06824j

**Published:** 2025-11-06

**Authors:** Mohammed Rehmanji, Alastair Skeffington, Karen A. Hudson-Edwards, Laura Newsome

**Affiliations:** a Camborne School of Mines, Environment and Sustainability Institute, University of Exeter Penryn Cornwall TR10 9FE UK m.rehmanji@exeter.ac.uk k.hudson-edwards@exeter.ac.uk lauranewsome1@gmail.com; b Department of Biological and Environmental Sciences, University of Stirling Scotland UK alastair.skeffington@stir.ac.uk

## Abstract

Microbial Induced Carbonate Precipitation (MICP) is a biogeochemical process that drives the formation of carbonate minerals. This study employed MICP, using the urease-overproducing bacterium *Sporosarcina pasteurii* to remove cationic metals from geothermal Li-bearing brines. MICP successfully removed 96% of Ca, 46% of Mg, 88% of Mn, and 91% of Sr from a natural brine solution. Over 96% of Li remained in the solution following the treatment process. Circular economy approaches were applied by using waste products to stimulate ureolysis and testing the slurry waste generated from bioprocessing of the brine for use as a soil amendment. *S. pasteurii* grew on 62.5 g L^−1^ of spent yeast extract and precipitated metal carbonates from natural brine at rates similar to those observed when cultivated in commercial media (TSB plus 30 g L^−1^ urea). *S. pasteurii* was also able to utilize urea from cow urine and precipitate an equivalent amount of calcium to commercial urea. The slurry was able to neutralize acidic soils and enhance the microbial activity of the soil. This study highlights the use of waste products (cow urine and spent yeast from the brewery industry) as cost-effective alternatives for the biomass production of *S. pasteurii*. The novelty of this study lies in the application of MICP using waste substrates, in the treatment of Li-bearing geothermal brines, and in demonstrating selective removal of scaling metals while retaining Li in solution, a significant step toward enabling efficient Li recovery.

## Abstract 2

Graphical representation of microbially induced carbonate precipitation by *Sporosarcina pasteurii* selectively removes divalent cations from geothermal brine, enabling sustainable lithium retention through a circular bioeconomy route.

## Graphical abstract

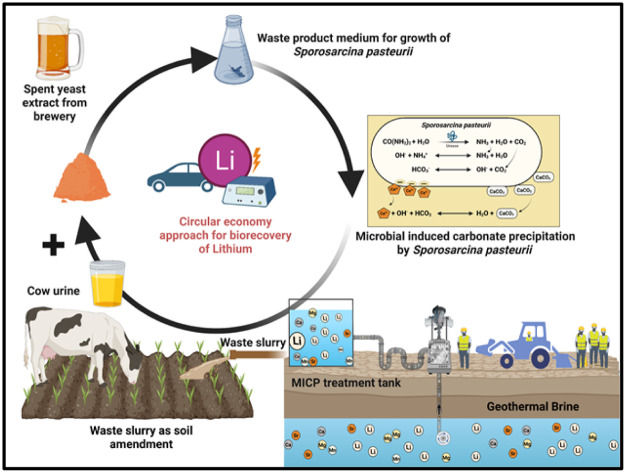

## 1 Introduction

Microbially-induced carbonate precipitation (MICP) has garnered attention for its potential in environmental applications such as aquifer decontamination,^1^ soil stabilization,^2^ carbon capture and storage,^3^ and coprecipitation of metals and radionuclides.^4^ MICP involves the biomineralization of calcium carbonate (CaCO_3_);^5^ urease catalyses the hydrolysis of urea, generating ammonium and bicarbonate, and rapidly raises pH. This provides favourable conditions for metal carbonate precipitation, possibly aided by electrostatic attraction of metal carbonates to the cell surface.^5^ This process is independent of bacterial growth as long as the cells contain active urease.

In parallel, circular economy approaches have emerged as a transformative framework aimed at decoupling economic growth from resource consumption.^6^ In a circular economy, waste from one process is repurposed as a valuable input for another, effectively reducing both environmental impacts and operational costs.^7^ This model has been successfully implemented in several bioprocess applications, including biogas production,^8^ bioleaching of metals,^9^ and the production of bioelectricity.^10^ Despite these, the application of circular economy strategies for Li recovery *via* MICP remains largely unexplored. In this study we aim to investigate circular economy approaches for MICP to selectively remove metal cations from Li-rich natural brine solution.

Lithium is becoming increasingly important due to its use in low carbon technologies^11^ and for energy storage.^12^ Global Li production has increased 1.5 fold from 2018 to 2022,^13^ yet projections indicate demand could surge 18–20-fold by 2050, creating substantial supply gaps.^14^ Adding to this challenge, the recent drop in lithium prices has led to the closure of several projects, further exacerbating the potential supply shortage in the future.^15^ As a result, companies are under growing pressure to explore lower-cost extraction methods to reduce operating expenses and strengthen their position on the cost curve, ensuring a sustainable supply of Li to meet future demand. Securing ample Li is imperative in the pursuit of Net Zero emissions by 2050.

Li reserves occur in different forms, including brine deposits (*e.g.*, Chile, Bolivia, Argentina), hard-rock ores (*e.g.*, Australia, China), as well as clay deposits (*e.g.*, USA, Mexico) and geothermal waters (*e.g.*, Germany, UK).^13^ Total Li reserves in brine (26.9 million tons) are higher than those in hard-rock ores (16.7 million tons).^16^ Li-bearing brines include those found in South American salt basins called salars and in geothermal environments.

Geothermal brines in fractured reservoirs within granitic rocks in Cornwall, UK,^17^ have been discovered to contain Li.^18^ Moreover, Salar de Atacama, Chile;^19^ Punta del Este Uruguay;^20^ Salton Sea known geothermal resource area (KGRA) California^21^ and the Upper Rhine Graben (URG),^22^ located in France and Germany border are recognized as high Li (>100 mg L^−1^) bearing geothermal brine locations worldwide.

Geothermal brines are primarily exploited as a source of thermal energy in power plants. Given their dual potential, it has been suggested that integrating Li extraction with heat recovery could significantly enhance the circular economy credentials of both geothermal power generation and Li production.^23^ This approach not only maximizes the economic value derived from geothermal resources but also contributes to sustainable and resource-efficient energy and material production. The technology to extract Li from brine primarily relies on open-air evaporation to concentrate the elements.^14^ However, this method leads to substantial water loss, raising concerns about its sustainability.^14^ Additionally, the inherently slow process of brine concentration *via* evaporation renders it unresponsive to short-term fluctuations in demand.

Among these, direct Li extraction (DLE) has emerged as a potential preferred option. DLE integrates various thermal and electrochemical processes, utilizing ion-exchange resins or sorption-based methods to extract lithium directly from brines.^24^ Despite the efficiency of DLE technologies, their integration into circular lifecycles is lacking, with no established industry-standard processing pipeline.

Plants processing geothermal brines, including *via* DLE technologies, are vulnerable to scaling and corrosion by the precipitation and attachment of solids to the surfaces of pipes and equipment can result in equipment fouling and subsequent reductions in brine production rates, plant flow rates, heat transfer efficiencies, and injection rates.^25,26^ The three most common scaling deposits in geothermal processing systems are silicon dioxide (silica), metal sulfides, and calcium bearing minerals.^27^ The primary focus of scale control efforts is usually Ca-bearing minerals which are typically present in much higher concentrations.^28^ Therefore, effectively targeting Ca removal from brines is an essential step in mitigating scale formation and reducing its associated problems. Failure to effectively manage scaling and corrosion issues can result in the shutdown of abstraction wells and surface facilities, driving up maintenance time and costs.^25,26^ Scale control programs usually rely on chemical inhibitors and acids, but the use of these harsh and often expensive chemicals poses significant environmental concerns.

Here we used MICP to selectively remove of metal cations from Li-rich natural brine solution using the urease producing bacterium *S. pasteurii*. We hypothesised that Li would remain in solution because Li carbonate has a much higher solubility product (*K*_sp_ 0.0218) compared to those of Ca and Mg carbonates (*K*_sp_ 3.3 × 10^−9^ and 6.82 × 10^−6^, respectively). Further integrating circular approaches into MICP offers a promising opportunity to enhance the efficiency of bioprocesses, thereby improving lithium recovery potential and contributing toward the development of more sustainable extraction workflows. Our research aimed to deliver an improved biotechnological process for Li recovery from geothermal brines based on circular economy principles.

## 2 Results and discussion

### 2.1 MICP in natural brine

**Fig. 1:**
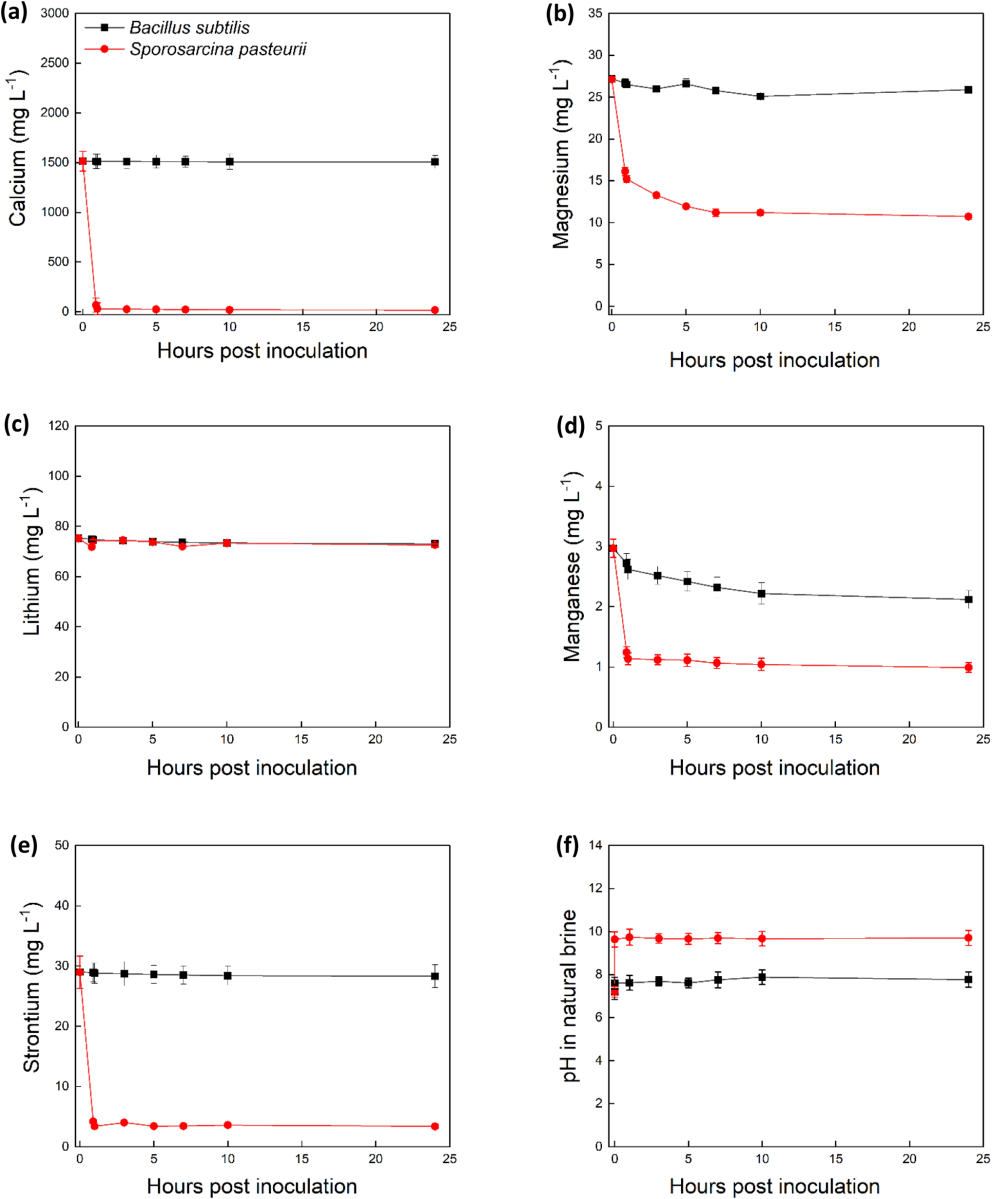
Microbial induced carbonate precipitation in natural brine inoculated with *S. pasteurii* (red circles) and *B. subtilis* (black squares). Plots show concentration of Ca (a), Mg (b), Li (c), Mn (d) Sr (e) and measured pH (f) in the solution over time. Error bars are the standard error of the mean, *n* = 3.

We hypothesized that MICP by *S. pasteurii* would remove metal cations from natural brine supplemented with urea. The results showed that while 96% of Li remained in solution, rapid decreases in Ca (96%), Mg (46%), Mn (88%), and Sr (91%) concentrations from natural brine were observed within the initial 10 minutes of the experiments (Fig. 1a, b, d and e), indicating rapid incorporation of these divalent cations into biogenic carbonates. This sharp initial decline coincided with a rapid pH increase (Fig. 1f), confirming the onset of MICP-driven carbonate precipitation. In contrast, 96% Li remained in the solution (Fig. 1c) highlighting the strong selectivity of the process toward lithium retention. Similar results were observed for all inoculum concentrations tested (Fig. S1–S4). The pH of the culture media increased from 7.2 to 9.4 during the first hour (Fig. 1f). As expected, no significant metal precipitation or changes in pH occurred with *B. subtilis* (control) cells that cannot perform ureolysis or in the abiotic control which contained urea in natural brine and no cells (Fig. 1 and S5).^29^ Based on these results the minimal initial optical density tested 0.05 at 600 nm was selected for further experimental analysis. Additional data on the metal content of natural brine (Table S1), growth rates of *S. pasteurii* and *B. subtilis* in TSB (Table S6), optical density and pH of the bacteria in TSB (Fig. S6a and b) are shown in SI. Minor discrepancies in ionic balance likely result from unmeasured species (*e.g.*, bicarbonate, fluoride, or organic acids) but do not compromise the core interpretations.

MICP has been extensively studied in soils, industrial wastewater, and freshwater systems,^30,31^ though its application in hypersaline environments has been largely overlooked. Table S8 compares the metal concentrations of various Li-bearing brines globally and the one used in this study. It shows that the Cornish brine is geochemically unique due to its high calcium, and moderate salinity levels. These conditions present a unique condition for MICP application which, represents as both a challenge and an opportunity: its high content of scaling ions complicates Li recovery, while its moderate Li concentration offers commercial value if selective pre-treatment (such as MICP) is applied. These features collectively position Cornish brine as an ideal candidate for biologically integrated mineral recovery systems and underscores the potential for MICP to be used in chemically diverse brine systems.

We compared MICP with a wide range of brine treatment strategies applied globally (Table S9). The MICP-based approach used in this study demonstrates exceptional efficiency and speed. Our study is the first to report removal of cations for Li recovery from geothermal brines using MICP. The performance far exceeds other reported methods, many of which require prolonged treatment durations or result in Li loss. Using MICP to selectively and rapidly remove competing metal ions, enhances the efficiency of Li recovery, a critical parameter in advancing direct lithium extraction (DLE) technologies. Moreover, the eco-friendly and scalable nature of MICP, positions this method as a highly promising platform for pre-treatment in Li recovery workflows.

### 2.2 Process characterization by XRD, PHREEQC and SEM analysis

The precipitates formed in the natural brine solution treated with *S. pasteurii* were identified using XRD as calcite, vaterite (both polymorphs of CaCO_3_) and magnesite (MgCO_3_) (Fig. S6). Although other metals were removed from the solution (Fig. 1), these were likely present in the solid phase at concentrations below the detection limit of XRD (5% by volume). The coexistence of these two polymorphs particularly the metastable vaterite has been widely associated with biologically induced mineralization, where microbial ureolysis locally alters pH and carbonate supersaturation conditions.^32^

**Fig. 2:**
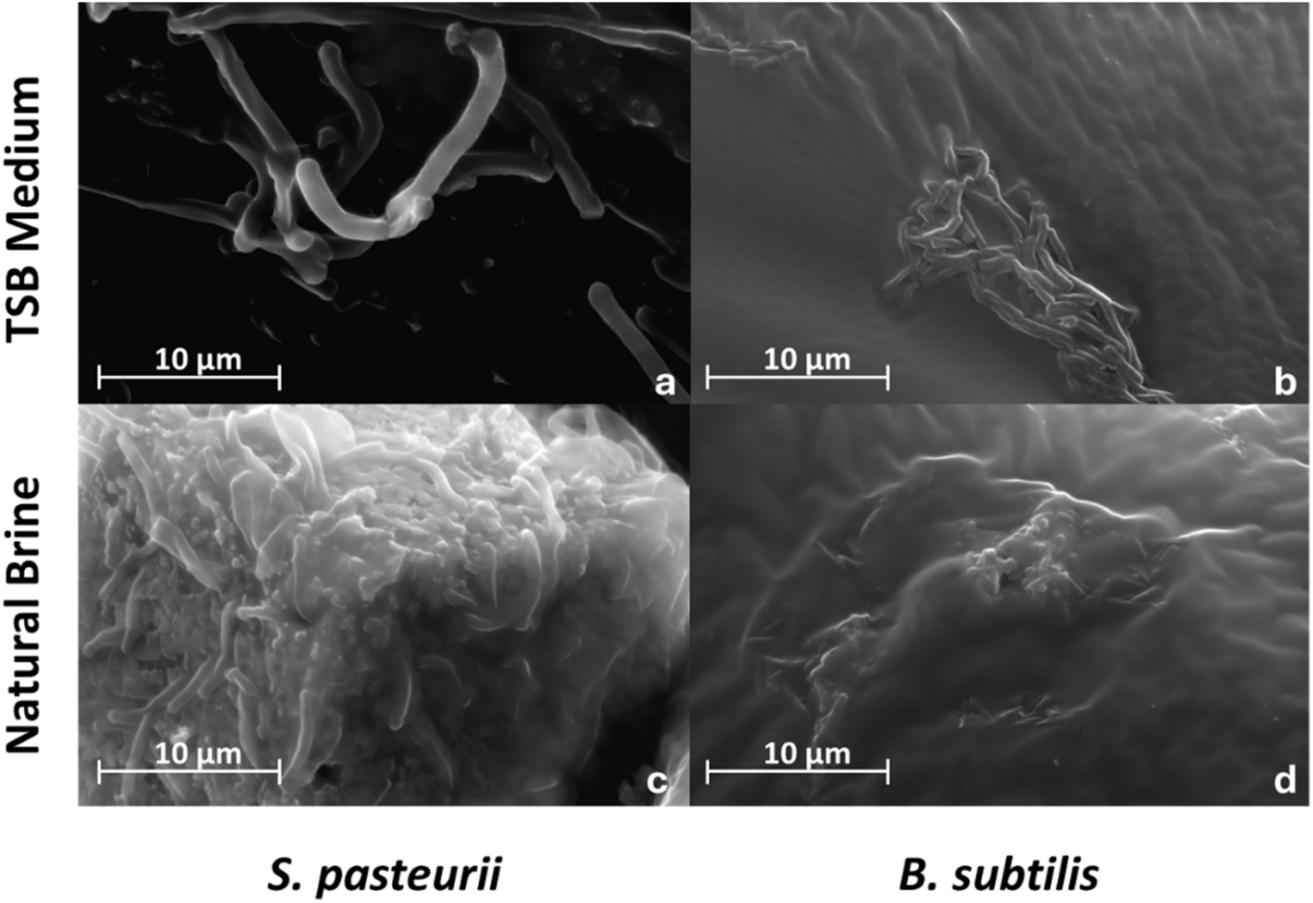
SEM photomicrograph showing bacterial cells and precipitates induced by the hydrolysis of urea by *S. pasteurii*: (a) intact *S. pasteurii* cells in TSB; (b) intact *B. subtilis* cells in TSB; (c) precipitate formed by *S. pasteurii* in natural brine solution, with bacteria embedded within growing precipitate crystals; (d) few intact cells of *B. subtilis* were found and no precipitate formed in natural brine. Images were acquired using an Everhart–Thornley detector (ETD) on the SEM operating, at an accelerating voltage of 25 kV, working distance of approximately 13 mm, magnification of 10^4^×, and a spot size of 5.0 nm.

Calcite precipitates formed were observed by SEM (Fig. 2) and exhibited the typical crystal morphology of bio-precipitated calcite.^33^ PHREEQC analysis suggested that calcite and vaterite were mostly supersaturated or near saturated in the natural brine solution (calculated saturation indices (SIs) of 0.09 to −1.03 and −0.69 to 0.44, respectively) (Table S7).

Weakly positive SIs were also calculated for dolomite (CaMg(CO_3_)_2_; 0.56–1.50) and magnesite (MgCO_3_; 0.02–0.10). Dolomite may have been present in small quantities that were undetectable by XRD. The precipitation of calcite, vaterite, and magnesite could explain the removal of Ca and Mg from natural brine (Fig. 1 and S7). The removal of Sr and Mn (Fig. 1d and e) may be due to incorporation of these metals in these minerals.^34–36^ The solubility products (*K*_sp_) of calcium carbonate (calcite and vaterite) and strontium carbonate are significantly lower than that of magnesium carbonate, indicating that Ca^2+^ and Sr^2+^ within these minerals likely to precipitate at the same carbonate concentration. Additionally, calcium and strontium readily participate in carbonate crystal nucleation and growth due to their lower hydration energies, which allows them to shed their hydration shells more easily and interact with carbonate ions.^37^ Magnesium, in contrast, has a high hydration energy and forms stable aqueous complexes (*e.g.*, Mg(H_2_O)_6_^2+^), its strong hydration shell hinders carbonate association making it kinetically less favourable for precipitation.

Overall, under MICP, when compared to Li, cations such as Ca^2+^, Mg^2+^, Sr^2+^, and Mn^2+^ readily precipitate as insoluble carbonates (*e.g.*, calcite, magnesite), owing to their low solubility products and stronger electrostatic interactions with carbonate species. In contrast, Li^+^, being monovalent and possessing a much higher solubility product for lithium carbonate (*K*_sp_ ≈ 2.18 × 10^−2^) along with a high hydration energy, does not readily form solid carbonate phases under the higher pH and carbonate concentrations achieved during MICP. Consequently, 96% of Li remains in the aqueous phase.

Previous studies have established that *S. pasteurii* serves as nucleation sites for calcium carbonate (CaCO_3_) precipitation in the MICP process.^38^ To verify this hypothesis, we examined the morphology of *S. pasteurii* and *B. subtilis* during MICP in natural brine using SEM (Fig. 2). *S. pasteurii* cultured in TSB medium exhibited perfectly intact cell forms (Fig. 2a). Following inoculation in natural brine, the *S. pasteurii* and *B. subtilis* cells retained their morphology and were embedded within the carbonate precipitate (Fig. 2c).

Notably, *S. pasteurii* maintained a highly intact cell structure even under mineralization condition. The negatively charged functional groups on the bacterial surface (*e.g.*, carboxyl and phosphate moieties) serve as active sites for cation adsorption and carbonate nucleation. No visible signs of precipitation were observed with the non-mineralizing bacterium *B. subtilis* (Fig. 2d). EDS data confirmed that the precipitate contained C, O, Mg, Ca, and Mn. Zeta potential measurements of *S. pasteurii* and *B. subtilis* supports the SEM analysis and are presented in the SI (Fig. S8). The robust cell structure of *S. pasteurii* suggests an adaptation mechanism to high pH and salt concentrations in natural brine.^39^ This structural integrity enhances the bacteria's role as a physical and chemical nucleation template, promoting localized supersaturation and mineral deposition without compromising cell stability.

### 2.3 Biomass production and MICP potential of *S. pasteurii* grown on cow urine

**Fig. 3:**
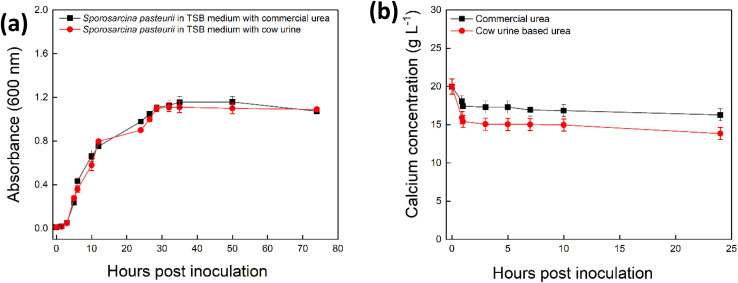
Growth performance and calcium precipitation of *S. pasteurii* in TSB medium with urea from cow urine (red circles) and with commercial urea (black squares). Plot show optical density measured at 600 nm in TSB medium (a), concentration of calcium in CaCl_2_ solution (b) over time. Error bars are the standard error of the mean, *n* = 3.

Cow urine is known to be a rich source of urea;^40^ samples obtained in this study contained 3 g L^−1^ (Fig. S9). There were no significant differences in growth curves of *S. pasteurii* in TSB medium with and without 3 g per L urea in the form of commercial urea or cow urine (Fig. 3a), indicating cow urine did not affect cell growth. Using cow urine to stimulate MICP showed that under non-growth conditions *S. pasteurii* precipitated slightly more Ca (4 g L^−1^) than commercial urea (6 g L^−1^) in the presence of equimolar amounts of urea from cow urine and commercial urea (Fig. 3b), demonstrating cow urine was successful as commercial urea in stimulating MICP.

The urea concentration of the unconcentrated cow urine was considerably lower (around 3 g L^−1^) than which can be achieved using commercial urea, *e.g.* the 30 g L^−1^ used in previous MICP experiments (Section 2.1), resulting in a reduced total Ca precipitation. Our cost analysis (Table 1) reveals that using cow urine reduces costs of *S. pasteurii* biomass production by 88% compared to commercial urea, making it economically attractive for large-scale applications. Secondarily factors such as animal diet, health, and collection practices can influence urea levels, potentially leading to inconsistencies in carbonate generation and precipitation efficiency. Therefore, while cow urine presents a sustainable and low-cost alternative, its large-scale use would benefit from simple standardization steps, such as batch pre-screening or concentration adjustments, to ensure consistent performance. Cow urine is already utilized successfully in various agricultural and traditional applications, demonstrating that standardization at scale is achievable,^41,42^ it has the potential to serve as a substitute for commercial urea, although further processing would be needed to concentrate the urine to achieve the urea concentrations equivalent to those used in typical MICP experiments.

Cost analysis of the ingredients used for cultivation of *S. pasteurii* in media with commercial standard nutrients and low-cost waste material. Commercial values for the medium components were obtained from Fisher Scientific, UK

**Table:** 

Ingredients of TSB medium	Concentration (g L^−1^)	Cost (£) TSB medium (per L)	Cost of producing 50 L biomass for treating 1000 L brines with urea (£)
Casien extract	17	1.7	85.0
SOYA extract	3	0.3	15.0
Glucose	2.5	0.2	10.0
Dipotassium hydrogen phosphate	2.5	0.2	10.0
Sodium chloride	5	0.5	25.0
Urea for biomass production	30	0.5	25.0
Urea for MICP in natural brine	30	0.0	500.0 (this is a cost for adding urea in natural brine and not for 50 L biomass production)
Total cost	NA	3.4	670.0

a There are likely to be additional costs associated with using cow urine such as such as collection, storage, and transport costs could influence large-scale applications which would need to be accounted for in a cost-benefit analysis for scaled up production, Future work could involve a more detailed assessment to quantify these factors and further evaluate the feasibility of waste-derived substrates in industrial MICP applications.

**Table:** 

Ingredients of waste product medium	Concentration (g L^−1^)	Cost (£) waste product medium (per L)	Cost of producing 50 L biomass for treating 1000 L brines with urea (£)
Yeast extract from brewery waste	62.5	0.0	0.0
Glucose	2.5	0.2	10.0
Dipotassium hydrogen phosphate	2.5	0.2	10.0
Urea for biomass production	3	0	0
Urea for MICP in natural brine	3	0	0
Total cost	NA	0.4	20.0^a^

### 2.4 Biomass production and MICP potential of *S. pasteurii* grown in SYE medium

**Fig. 4:**
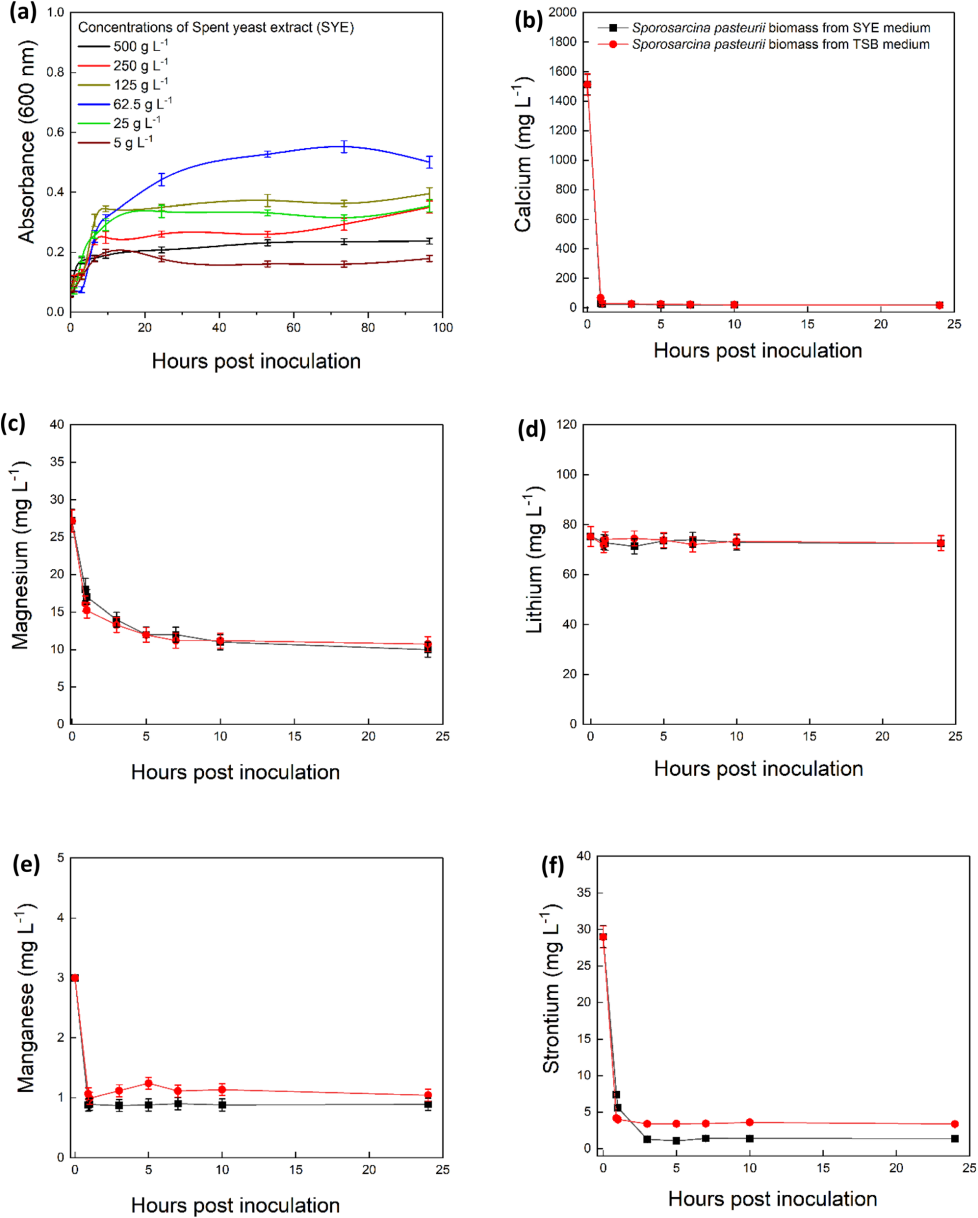
Microbial induced carbonate precipitation potential in natural brine with *S. pasteurii* cultivated in 62.5 g per L spent yeast extract (SYE) medium (black square) and 30 g per L TSB medium (red circles) prior to inoculation in natural brine. Plots show growth of *S. pasteurii* in spent yeast extract medium (a), concentrations of Ca (b), Mg (c), Li (d), Mn (e) and Sr (f) in the natural brine over time. Error bars are the standard error of the mean, *n* = 3.

Spent yeast extract was tested as an alternative to TSB medium for growing *S. pasteurii*. The maximum O.D. (0.55) for *S. pasteurii* grown in 5–500 g L^−1^ of SYE was recorded in 62.5 g per L SYE and the minimum O.D. (0.16) was recorded in 5 g L^−1^ of SYE (Fig. 4a). To determine whether cells grown on SYE could be used for MICP to treat Li-bearing natural brine, *S. pasteurii* grown in 62.5 g L^−1^ of SYE was inoculated in a natural brine solution supplemented with 500 mM urea. The amounts and rates of metal precipitation from these SYE experiments (Fig. 4b and f) were similar to those of *S. pasteurii* cells grown on commercial media. This indicates that the SYE can serve as an alternative growth medium for MICP and potentially be applied for the scale-up bioprocessing of Li bearing brine solution.

To assess the financial benefits of using waste products for MICP, costs were calculated for purchasing the components required using commercially available substrates, and for a medium based on freely available waste products (spent yeast extract and cow urine). According to 2024 prices the components for the waste product medium cost £20 per 1000 L and for the commercially available substrate cost £670 per 1000 L (Table 1). Therefore, the cost could be reduced by 88% for *S. pasteurii* biomass production and by 96% for the 1000 L of natural brine bioprocessing. These significant cost reductions underscore the economic advantages of incorporating waste-derived substrates into MICP processes, contributing to a circular economy and reinforcing the potential for scalable and sustainable biotechnological applications.

### 2.5 Evaluating the effect of slurry addition on soil health

The bioprocessing of Li bearing brines at a large scale is likely to generate substantial quantities of slurry, rich in CaCO_3_, MgCO_3_, Sr and Mn, along with residual bacterial cells. This slurry has the potential to serve as a soil amendment, contributing to nutrient enrichment and organic matter input. After addition of TSB MICP slurry, the soil (from research field in Cornwall) pH increased from 4.5 to 7.6, and from 4.5 to 7.3 after SYE MICP slurry addition. The soil from garden field in Cornwall also showed an increased in pH from 5.9 to 7.3 after TSB MICP slurry addition and from 5.9 to 7.8 after SYE MICP slurry addition (Table 2). Lime is widely recognized as a natural soil conditioner that corrects soil acidity, increases nutrient availability, and improves soil structure.^43^ However, lime is one of the more expensive farm inputs.^43^ Soil analysis indicated an increase in pH following the addition of the waste slurry (Table 2), and XRD analysis confirmed that the precipitates in the slurry are identified as calcite, vaterite, and magnesite (Fig. S6).

Parameters depicting soil health after treating 500 g of each soil type with different slurries. The experiment included six samples with the control samples having no addition of slurry: (1) research field soil (control), (2) garden soil (control), (3) research field soil + slurry generated after treating natural brine with *S. pasteurii* grown on TSB medium, (4) research field soil + slurry generated after treating natural brine with *S. pasteurii* grown on SYE medium, (5) garden soil + slurry generated after treating natural brine with *S. pasteurii* grown on TSB medium, and (6) garden soil + slurry generated after treating natural brine with *S. pasteurii* grown on SYE medium. Error bars are the standard error of the mean, *n* = 3. Friendly, economical and scalable. Further repurposing the slurry waste (Table 1) generated

**Table:** 

Samples	pH	P content (mg L^−1^)	K content (mg L^−1^)	Mg content (mg L^−1^)
Research field soil (control)	4.5 ± 0.4	66.4 ± 1.3	246 ± 2.2	190 ± 1.3
Garden soil (control)	5.9 ± 0.7	29.4 ± 1.2	100 ± 1.3	101 ± 2.1
Research field soil + TSB slurry	7.6 ± 0.1	54.2 ± 2.2	263 ± 2.1	106 ± 2.3
Research field soil + SYE slurry	7.3 ± 0.2	58.4 ± 1.2	251 ± 1.4	119 ± 1.5
Garden soil + TSB slurry	7.3 ± 0.3	39.4 ± 1.3	142 ± 1.2	99.1 ± 1.3
Garden soil + SYE slurry	7.8 ± 0.1	41.0 ± 1.2	146 ± 1.1	74.1 ± 1.1

**Table:** 

Samples	Organic matter (%)	Soil respiration (CO_2_ evolution in mg kg^−1^)	Neutralizing value of soil equivalent to calcium carbonate (CaCO_3_) (% w/w)	Neutralizing value of soil equivalent to calcium oxide (CaO) (% w/w)
Research field soil (control)	11.3 ± 0.1	24.0 ± 1.3	<1	<1
Garden soil (control)	15.9 ± 0.1	184 ± 1.1	<1	<1
Research field soil + TSB slurry	10.5 ± 0.3	122 ± 1.2	1.6 ± 0.1	<1
Research field soil + SYE slurry	10.9 ± 0.1	119 ± 1.3	1.8 ± 0.2	1.0 ± 0.2
Garden soil + TSB slurry	16.6 ± 0.2	209 ± 2.2	2.2 ± 0.3	1.3 ± 0.1
Garden soil + SYE slurry	14.4 ± 0.3	197 ± 0.5	2.4 ± 0.1	1.4 ± 0.1

The nutrient profile of the soil demonstrates that phosphorus (P) concentrations increased from 29 mg L^−1^ to 41 mg L^−1^ and potassium (K) concentrations increased from 100 mg L^−1^ to 147 mg L^−1^ in garden soil after the SYE slurry addition. Similar results were obtained with this soil after TSB slurry addition (Table 2). By contrast no considerable changes in P and K concentration were recorded for the research field soil after slurry addition. Mg soil concentrations decreased after all slurry additions, possibly due to dilutions caused by the slurries (Table 2). Slurry treatment did not affect the organic matter content of the soils, but it increased their CO_2_ evolution which is directly related to soil respiration, a general measure of biological activity, indicating microbial biomass, carbon sequestration, and nitrogen mineralization rates (Table 2).^44^ In the control research field soil, the CO_2_ evolution was 24 mg kg^−1^, whereas after slurry addition it reached 122 mg kg^−1^. Similarly, slurry addition to the garden soil, increased CO_2_ evolution from 184 mg kg^−1^ to 209 mg kg^−1^. While the increase in soil pH and microbial respiration following slurry addition demonstrates its agronomic potential, long-term effects were not evaluated in this study. The slurry Mn (2.6 mg L^−1^) and Sr (26.4 mg L^−1^) incorporated within carbonate phases, which under certain environmental conditions could potentially undergo dissolution or leaching. However, these elements are both naturally present in soils, and the concentrations in slurry are low compared to average background concentrations of 240 mg per kg Sr^45^ and 330 mg per kg Mn^46^ (ATSDR toxicological profiles).

However, given their low solubility as carbonates under the neutral to alkaline pH observed post-application, immediate risks of metal toxicity are expected to be minimal. Future work could include long-term leaching experiments and monitoring of Mn and Sr mobility to ensure environmental safety and stability of the treated soils. Repurposing the slurry waste (Table 2) generated during the bioprocessing as a soil amendment, enhances resource utilization and environmental benefits.

## 3 Conclusions

Our experimental results confirm that MICP can effectively remove unwanted cations while leaving 96% Li in the solution, suggesting its compatibility with existing extraction frameworks. The preferential removal of certain cations results from a combination of lower solubility products, more favourable nucleation kinetics, and stronger microbial surface interactions. These factors collectively lead to efficient precipitation of metal carbonates in the MICP process. The potential to couple MICP with direct lithium extraction (DLE) technologies offers a promising pathway for developing more sustainable, time-efficient, cost-effective and universal strategies for Li brine processing supporting the advancement of green technologies and responsible mineral resource recovery. Moreover, the process was successfully integrated with waste-derived substrates such as spent yeast extract and cow urine, demonstrating the integration of circular economy principles. The slurry produced after brine treatment could serve as a cost-effective alternative to conventional lime in agricultural soils. Collectively, these results underscore the broader implications of integrating circular economy principles into MICP.

## 4 Materials and methods

### 4.1 Microorganism and growth conditions

*Sporosarcina pasteurii* DSM33 was obtained from the Leibniz Institute, DSMZ German collection of microorganism and cell culture and *Bacillus subtilis* ATCC-2385 was obtained from the American Type Culture Collection (ATCC), UK as a urease-negative control.^47^ Both strains were cultured in tryptone soy broth (TSB) (30 g L^−1^) with added urea (Thermo Fisher Scientific, UK). The TSB medium at pH ≈ 7.4 was sterilized by autoclaving at 121 °C for 20 min. A 10 M urea stock solution was filter-sterilized (PTFE syringe filters, pore size 0.2 μm, Thermo Fisher Scientific, UK) and separately added to the TSB medium at a final concentration of 500 mM before bacterial inoculation.

Both bacterial cultures were defrosted from −80 °C and plated on TSB agar plates (30 g per L TSB, 1.5 g per L agar, 30 g per L urea); for *B. subtilis* cultures urea was excluded from the agar plate. Single colonies were taken and inoculated in 15 mL tubes (TSB + urea) and kept overnight. These were considered as primary cultures to inoculate larger culture volumes, which were grown to mid-log phase before being used to initiate MICP experiments. Secondary cultures (250 mL flasks with 100 mL culture medium, TSB + urea) were kept for overnight growth, at 28 °C, 180 rpm in an orbital type Infors HT Multitron PRO Triple Stack Incubator Shaker (UK) for all the experiments. The growth of *S. pasteurii* and *B. subtilis* was monitored in standard plastic cells by measuring the optical density at 600 nm with a Multiskan SkyHigh Microplate Spectrophotometer (Thermo Scientific, UK) and mid-logarithmic phase cells selected to start the MICP experiments. The cell growth was calculated using following equation *μ* = ln(*N*_2_/*N*_1_)/(*t*_2_ − *t*_1_) where *μ* is the specific growth rate and *N*_1_ and *N*_2_ are the optical densities at times (*t*_1_ and *t*_2_), respectively. Doubling time was also calculated by the following equation: doubling time = ln(2)/*μ*.^48^

### 4.2 Natural brine and MICP experiments

To test the hypothesis that MICP could be used to remove metal cations from Li-bearing natural brine and to evaluate the optimal cell concentration required for performing MICP, natural geothermal brine was obtained from a Cornish geothermal project (Cornwall, South-West England, UK). The composition of the brine was determined (Section 4.4) and results are given in Table S1. Mid-logarithmic phase *S. pasteurii* cells from the secondary culture were harvested by centrifugation (3200*g*, 15 minutes) and washed using phosphate buffered saline, centrifuged again, and inoculated into the natural brine solution with varying initial optical densities (0.05, 0.1, 0.2, 0.3, and 0.4), in standard 250 mL conical flasks containing 100 mL medium. The brine was not sterilized before the experiments as these were conducted under non-growth conditions with large amounts of biomass and no nutrients added, to ensure the experiments represented the conditions of a scaled-up industrial process. To stimulate ureolysis and MICP, 500 mM of urea was added, and flasks were incubated at 28 °C in an Infors HT Multitron PRO (UK) incubator without shaking for 24 h. The urease negative control was *Bacillus subtilis*. Aliquots were collected for geochemical characterization after 0, 1, 3, 5, 7, 10, and 24 hours. The experiments were conducted in triplicate.

### 4.3 Using waste products (spent yeast extract and cow urine) in the MICP process

The ability of *S. pasteurii* to grow in media created from waste products was tested with brewery waste and cow urine. Cells were grown in different concentrations of spent yeast extract (SYE) obtained from Verdant Brewing Co., Ltd. (Cornwall, UK). The SYE was sonicated for 30 min and autoclaved at 121 °C for 20 min to homogenize the extract, facilitating uniformity and nutrient availability, and used to make a low-cost medium comprising 2.5 g per L glucose, 2.5 g per L dipotassium hydrogen phosphate, and SYE at 5, 25, 62.5, 125, 250 and 500 g L^−1^. The concentration range was selected from previous literature studies.^49,50^ The medium was inoculated with mid-logarithmic phase *S. pasteurii* cells from the secondary culture (post-washing using phosphate buffer saline) at an O.D. of 0.05 in a standard 250 mL conical flasks containing 50 mL of medium. The experiments were incubated at 28 °C for 96 h with shaking (180 rpm) in an orbital type Infors HT Multitron PRO Triple Stack Incubator Shaker (UK) and monitored at regular intervals for growth *via* O.D_600_ nm. Cells grown at the optimal concentration of SYE (62.5 g L^−1^) were selected for testing their MICP potential. The *S. pasteurii* cells were harvested by centrifugation (3200*g* for 15 minutes), washed using PBS, and inoculated in natural brine (added with 500 mM urea) at a final O.D. of 0.05.

To evaluate cow urine as a potential source of urea for MICP, samples were sourced from Roscrowgney Farm (Cornwall, UK). The fresh cow urine was sterilized using 0.22 μm filters and stored at 4 °C to avoid degradation before the experiments commenced. The concentration of urea was quantified using a colorimetric assay (see Section 4.4). To evaluate the impact of cow urine on the growth of *S. pasteurii*, 30 g of TSB was added per L of cow urine. Bacterial cultures were inoculated from secondary cultures as above at a final OD_600_ of 0.05 into cow urine supplemented TSB medium. Experiments were incubated at 28 °C for 96 h with shaking (180 rpm) and growth was monitored at regular intervals *via* OD_600_ nm for 24 hours. For testing the MICP potential of *S. pasteurii* with cow urine as the source of urea, a synthetic solution of CaCl_2_ (20 g L^−1^) was prepared in cow urine and *S. pasteurii* cells were inoculated at a final O.D. of 0.4. The experimental control was a 20 g per L CaCl_2_ solution containing an equivalent concentration of commercial urea to the amount of urea present in cow urine. Flasks were incubated at 28 °C in the incubator without shaking for 24 h. Samples were collected for urea and calcium analysis after 0, 1, 3, 5, 7, 10, and 24 hours. All experiments were conducted in triplicate.

### 4.4 Chemical analysis

Concentrations of Na, K, Li, Ca, Mg, Sr, B, Cu, Fe, Mn and Ni in the natural brine solution were determined using an Agilent 5110 VDV Inductive Coupled Plasma-Optical Emission Spectrometer (ICP-OES). The brine solution used for ICP-OES was diluted (1 : 1000) into 2% nitric acid (HNO_3_). All reagents used were analytical grade. Elemental measurements were made in axial view mode using wavelengths and operating parameters are presented in the Table S2.

For anion analysis the natural brine was passed through 0.22 μm filters, diluted using deionized water and analysed using a high-pressure ion chromatography system (Dionex Integrion HPIC, Thermo Fisher Scientific), equipped with a Dionex IonPac AS11-HC column (2 mm × 250 mm, 4 μm particle size) and a 10 μL injection loop was employed. Chromeleon 7.0 software was used for data processing and anion concentrations were measured using standard calibrations. The instrumental parameters are listed in Table S3.

Urea concentrations were estimated using spectrophotometric assay as described by Zawada *et al.*^51^ and modified according to Rehmanji *et al.*^52^ Briefly, experimental samples at different time points were centrifugation (3200 × *g* for 15 minutes), 50 μL of supernatant was transferred into a clear flat-bottom 96-well plate. The working reagent included *o*-phthalaldehyde (100 mg L^−1^), primaquine bisphosphate (513 mg L^−1^), sulfuric acid (2.5 M), boric acid (2.5 g L^−1^), and Brij-35 (0.03%). Two hundred microliters of freshly prepared working reagent were added to the supernatant and mixed gently on a shaker for 1 h at 20 ± 2 °C. The optical density at 430 nm was measured and results were compared to a calibration curve made using 50 mg L^−1^ of commercial urea. Experimental pH and conductivity were measured with a pH meter S400 (METTLER TOLEDO UK) and conductivity meter AB330 (Thermo Scientific USA) using calibrated electrodes. Calibration was performed before analysis using standard buffer solutions.

### 4.5 Scanning electron microscopy (SEM)

To characterize the minerals formed at the end of the MICP experiments, the solid precipitates and cell mixture was centrifuged (3200*g*, 15 minutes). Two mL of cells and solid precipitate was collected and washed three times with sterile deionized water. After washing the pellet was fixed in 1 mL of 2.5% glutaraldehyde solution overnight and then centrifuged (3200*g*, 5 minutes) to remove the residual glutaraldehyde. The samples were further treated with 1 mL of 1.5% glutaraldehyde for 30 min, centrifuged (3200*g*, 15 minutes) and finally treated with 1 mL of 0.75% glutaraldehyde as described in ref. 53 Subsequently, cells were sequentially dehydrated with ethanol (30%, 40%, 50%, 60%, 70%, 80%, 90% and 100%) for 30 min for each concentration. The 100% ethanol samples were loaded on clean silicon wafers attached to aluminium stubs and left to dry in air. The images of the samples were obtained using a FEI Quanta FEG 650 SEM-EDS. EDS analysis was performed at multiple locations on each sample to confirm the distribution and relative abundance of key elements within the mineral matrix. EDS spectra and corresponding elemental maps were collected to identify elements characteristic of minerals associated with MICP, providing a comprehensive understanding of elemental associations and potential biomineralization pathways. The operating parameters are described in Table S4.

### 4.6 X-ray diffraction (XRD)

The mineralogy of the solid precipitates from the treated brine solution was determined with X-ray diffraction spectroscopy (XRD). To obtain 1 g of dry powdered cell solid precipitate pellet, 15 mL sample was collected at 24 h, centrifuged (3200*g* 15 min) and the pellet was dried at 60 °C for 24 h. The dry powdered sample was pressed into a plastic sample holder using a glass slide and made smooth, flat, and level. The sample was analysed using a Siemens D5000 XRD, (see Table S5 for details). Using the EVA v.18.0.0.0 software the JCPDS PDF-2 (2004) database was used to match peaks produced by the sample scan with known mineral *d*-spacings.

### 4.7 PHREEQC

To determine the phases of formed in the precipitates, speciation-solubility calculations were conducted using the PHREEQC code.^54^ Input data included experimental pH and temperature conditions and the measured aqueous concentrations. The pitzer.dat^55^ thermodynamic database for brines distributed with the code was used in the modelling. Thermodynamic data for vaterite were added to the Pitzer database to allow for determination of its saturation indices.

### 4.8 Zeta potential measurement

The electrophoretic mobility and zeta potential of bacterial cells were measured using a Zetasizer Nano Z instrument (Malvern Instruments, United Kingdom). To capture change in the cell surface charge in different solutions, *S. pasteurii* and *B. subtilis* were cultivated in tryptic soy broth (TSB) containing 500 mM urea. For measuring the change in the cell surface charge at different pH, 50 mL of *S. pasteurii* and *B. subtilis* cultures (OD_600_ ≈ 0.5) were placed inside a magnetically stirred beaker individually. 0.25 M HCl and 0.25 M NaOH were used to adjust the pH to the range required for zeta potential measurement (pH 2–11). Further samples were transferred into DTS1070 disposable folded capillary cells for analysis. Zetasizer software 8.01 (Malvern) was used for data processing, and the zeta potential was measured using a manual mode of operation.

### 4.9 Soil analysis

To assess the impact of incorporating waste slurry generated after brine treatment into agricultural soils, we conducted soil health analyses were conducted on two distinct soil types. Soil was collected from a research field located at the University of Exeter Penryn Campus (50°10′ N, 5°8′ W)′ and garden located at the Camborne, UK (50°16′ N, 5°2′ W). These two samples without addition of waste slurry formed the controls for this study. 0.1 L of slurry was obtained from the treatment of 2 liter of natural brine solution treated with *Sporosarcina pasteurii* grown in TSB medium and the SYE medium. Post-slurry addition, soil samples were sent for soil health analysis to NRM commercial laboratories (UK).

#### 4.9.1 Cost comparison analysis between commercial medium and waste product medium

Tryptone soy broth media was selected and used in this study as commercial medium for cost comparison with the waste product media which include spent yeast extract and cow urine-based urea. An arithmetic approach was employed to compare the costs of commercial media and the waste product medium. Price data for each component of the commercial medium were obtained from standard suppliers, while cost information for the raw materials used in the waste product medium was similarly collected. The cost per L of each medium was calculated to compare the cost between the two media formulations (Table 1).

## Author contributions

Mohammed Rehmanji: methodology, investigation, analysis, writing – original draft. Laura Newsome: supervision, methodology, conceptualization of idea, review & editing, funding acquisition, validation, writing, review & editing, project administration. Karen Hudson-Edwards: supervision, PHREEQC analysis, methodology, investigation, writing, review & editing, funding acquisition, validation, project administration. Alastair Skeffington: conceptualization of idea supervision, funding acquisition, review & editing.

## Conflicts of interest

The authors declare that they have no known competing financial interests or personal relationships that could have appeared to influence the work reported in this paper.

## Supplementary Material

RA-015-D5RA06824J-s001

## Data Availability

All data supporting the findings of this study are provided in the manuscript and the accompanying supplementary information (SI). No additional datasets were generated or deposited. Supplementary information is available. See DOI: https://doi.org/10.1039/d5ra06824j.
