# Supplementary material for: Circular economy approaches to microbially-induced carbonate precipitation for bioprocessing of geothermal brine for lithium recovery
Source: RSC Adv. 2025 Nov 6;15(51):43263–74. doi: 10.1039/d5ra06824j (PMC12590475; doi:10.1039/d5ra06824j)
Supplement: RA-015-D5RA06824J-s001 [file RA-015-D5RA06824J-s001.pdf]

Supplementary Information (SI) for RSC Advances.
This journal is © The Royal Society of Chemistry 2025

SUPPORTING INFORMATION

Circular economy approaches to microbially-induced carbonate precipitation for bioprocessing of geothermal brine for lithium recovery

Mohammed Rehmanji. *[1], Alastair Skeffington.[2], Karen A. Hudson-Edwards.[1], and Laura Newsome.[1,]

[1] Camborne School of Mines & Environment and Sustainability Institute, University of Exeter, Penryn TR109FE, Cornwall, United Kingdom.

[2] Department of Biological and Environmental Sciences, University of Stirling, Scotland United Kingdom.

***Corresponding Author and Present Address**

Mohammed Rehmanji, Camborne School of Mines & Environment and Sustainability Institute, University of Exeter, Penryn TR109FE, Cornwall, United Kingdom.

E-mail: m.rehmanji@exeter.ac.uk; Phone - +447448603289

Table S1. Chemical composition (mg L^{-1}) of natural brine used in this study. Errors are the standard error of the mean, n = 3.

Parameters/Cations/Anions	Natural Brines
**T (°C)**	28.0 ± 2.3
**pH**	7.2 ± 0.5
**EC (mS)**	20.3 ± 1.5
**Silica (mg L^{-1})**	35.0 ± 2.0
**Calcium (mg L^{-1})**	1512.0 ± 2.4
**Magnesium (mg L^{-1})**	27.2 ± 1.3
**Potassium (mg L^{-1})**	204.9 ± 3.3
**Sodium (mg L^{-1})**	2351.0 ± 3.2
**Lithium (mg L^{-1})**	75.2 ± 1.8
**Boron (mg L^{-1})**	12.8 ± 1.3
**Copper (mg L^{-1})**	2.0 ± 0.5
**Iron (mg L^{-1})**	2.5 ± 0.8
**Manganese (mg L^{-1})**	3.0 ± 0.4
**Strontium (mg L^{-1})**	29.0 ± 0.8
**Nickel (mg L^{-1})**	2.5 ± 0.9
**Chloride (mg L^{-1})**	7711.0 ± 4.5
**Sulphate (mg L^{-1})**	270.0 ± 5.5
**Phosphate (mg L^{-1})**	141.9 ± 8.7

Table S2. Operating conditions of Inductive coupled plasma optical emission spectroscopy (ICP-OES) and wavelengths at which the metals were detected.

**Wavelengths and operating conditions used for ICP-OES determination of metals.**	
**RF power (kW)**	1.2
**Plasma gas flow (L min^{-1})**	12
**Auxiliary Ar (L min^{-1})**	1.0
**Nebulizer Ar (L min^{-1})**	0.70
**Pump rate (mL min^{-1})**	1
**Readings/replicate**	3
**Wavelength (nm)**	Ca: 422.673; Mg: 285.213; Cu: 327.395; Fe: 238.204; Ni: 230.299; Mn: 294.921; Sr: 421.552; K: 769.897; B: 249.678; Na: 588.995; Sr: 421.552

Table S3. Operating conditions of Ion chromatography (IC) for anion detection in Natural brine.

**Operating conditions used for IC determination of anions.**	
**Eluent KOH (mM)**	30
**Operation mode**	Isocratic
**Flow rate (mL min $^{-1}$)**	0.38
**Suppressor (mA)**	29
**Column temperature (°C)**	30
**Pressure (psi)**	< 4000

Table S4. Operating conditions of scanning electron microscopy (SEM).

Operating conditions used for SEM analysis.	
**Accelerating voltage (kV)**	25
**Working distance (mm)**	20
**Electron beam current (µA)**	233
**Magnification (X)**	5 - 10^5
**Chamber pressure (Pa)**	10
**Interpretation software**	xT microscope control 6.2.7

Operating conditions used for XRD analysis.	
**XRD**	Siemens D5000 diffractometer
**X-Ray Tube**	1.5kW, Cu-anode
**kV & mA**	40kV, 30mA
**Wavelength**	1.5406/1.54439 (Cu Kα1/2)
**Detector**	Scintillation point detector
**Div. Slit**	V6 (variable, 6mm)
**AntiScatter Slit**	(same as above)
**Scan Type**	θ/θ locked
**Scan Range**	2-70 °2θ
**Step Size**	0.02 °2θ
**Scan time**	1 sec/step
**Scan Rotation**	Yes
**Interpretation software**	EVA v.18.0.0.0.
**PDF Database**	JCPDS PDF-2 (2004) database

Table S5. Operating conditions of X-ray diffraction analysis.

Table S6. Growth performance of *S. pasteurii* and *B. subtilis* in tryptone soy broth in exponential phase, represented in terms of growth rate (h^{-1}), doubling time (h), maximum optical density (O.D. @ 600 nm) and pH of medium. Errors are the standard error of the mean, n = 3.

Parameters/Organisms	***Bacillus subtilis*** **in tryptone soy broth**	***Sporosarcina pasteurii*** **in tryptone soy broth**
**Growth rate (h^{-1})**	0.69 ± 0.05	0.71 ± 0.03
**Doubling time (h)**	0.99 ± 0.01	0.97 ± 0.02
**Maximum OD_{600}**	1.15 + 0.04	1.16 ± 0.05
**Maximum pH**	7.37 ± 0.06	9.60 ± 0.03

Table S7. Saturation indices calculated for minerals formed via MICP in the natural brine solution.

Sample	Time point	Calcite	Aragonite	Dolomite	Huntite	Magnesite	Vaterite
Bacillus subtilis **in Natural brine solution**	0	0.51	0.22	-0.48	-5.51	-1.76	-0.09
Bacillus subtilis **in Natural brine solution**	0.9	0.75	0.46	0.01	-4.52	-1.51	0.15
Bacillus subtilis **in Natural brine solution**	3	0.88	0.58	0.21	-4.17	-1.44	0.28
Bacillus subtilis **in Natural brine solution**	7	0.71	0.42	-0.12	-4.84	-1.61	0.31
Bacillus subtilis **in Natural brine solution**	24	0.91	0.62	0.28	-4.05	-1.41	0.31
Sporosarcina pasteurii **in Natural brine solution**	0	0.41	0.12	-0.68	-5.91	-1.86	-0.18
Sporosarcina pasteurii **in Natural brine solution**	0.9	0.92	0.63	1.41	-0.68	-0.29	0.33
Sporosarcina pasteurii **in Natural brine solution**	3	0.13	-0.16	0.32	-2.34	-0.58	-0.46
Sporosarcina pasteurii **in Natural brine solution**	7	0.03	-0.26	0.20	-2.51	-0.6	-0.56
Sporosarcina pasteurii **in Natural brine solution**	24	-0.09	-0.39	0.07	-2.65	-0.61	-0.69

Table S8. Composition of different lithium-rich brines reported from literature.

Deposit Country

Cations/ Anions	Calcium (mg L^{-1})	Magnesium (mg L^{-1})	Potassium (mg L^{-1})	Sodium (mg L^{-1})	Lithium (mg L^{-1})	Boron (mg L^{-1})	Chloride (mg L^{-1})	Sulphate (mg L^{-1})
**Uyuni (Bolivia)**	460	7680	7920	98400	320	190	177600	12960
**Atacama (Chile)**	450	9650	23600	91000	1570	440	189500	15900
**Olaroz (Argentina)**	1100	2000	5300	111500	570	NA	177500	9800
**Hombre Muerto (Argentina)**	120	140	9700	103000	900	540	168000	11400
**Zabuye Lake (China)**	0	0	45960	127900	660	1580	147600	26290
**Clayton Valley (USA)**	450	230	8000	63700	360	90	100000	6600
**Taijinar Salt Lake brine (China)**	300	13500	NA	102300	210	310	188100	24000
**Southern Tibet geothermal belt (China)**	50	20	NA	1700	130	NA	2000	1700
**Chott Djerid (Tunisia)**	1600	3400	5600	80000	60	NA	144100	6700
**Cerro prieto (USA)**	30800	3200	23500	60000	250	500	175500	46000
**Salton Sea (USA)**	25700	110	14500	49200	200	300	142000	100
**Rincon (Argentina)**	600	3000	6600	97900	300	400	158000	NA
**Soultz-sous-Forêts (France)**	7200	130	3200	28100	170	40	58600	160
**Buhl geothermal well (Germany)**	11600	1930	490	64000	40	NA	120300	1600
**Landau geothermal well (Germany)**	7700	80	4000	28200	180	NA	64200	100

	**Dieng geothermal power plant (Indonesia)**	400	3	2200	39	40	300	13600	3
	**Coipasa (Bolivia)**	NA	13600	11000	75100	350	800	151000	24600
	**Cauchari (Argentina)**	NA	1450	4200	93300	510	1100	148600	15700
**Current study**	**Cornwall (United Kingdom)**	1512	27.2	204.9	2351	75.2	12.8	7711	270

	**Cations/ Anions**	**Copper (mg L^{-1})**	**Iron (mg L^{-1})**	**Manganese (mg L^{-1})**	**Strontium (mg L^{-1})**	**Nickel (mg L^{-1})**	**Phosphate (mg L^{-1})**	**Silica (mg L^{-1})**	**References**
**Deposit Country**	**Uyuni (Bolivia)**	NA	NA	NA	NA	NA	NA	NA	[1]
	**Atacama (Chile)**	NA	NA	NA	NA	NA	NA	NA	[1]
	**Olaroz (Argentina)**	NA	NA	NA	NA	NA	NA	NA	[2]
	**Hombre Muerto (Argentina)**	NA	NA	NA	NA	NA	NA	NA	[1]
	**Zabuye Lake (China)**	NA	NA	NA	NA	NA	NA	NA	[1]
	**Clayton Valley (USA)**	NA	NA	NA	NA	NA	NA	NA	[1]
	**Taijinar Salt Lake brine (China)**	NA	NA	NA	NA	NA	NA	NA	[3]
	**Southern Tibet geothermal belt (China)**	NA	NA	NA	NA	NA	NA	NA	[3]
	**Chott Djerid (Tunisia)**	NA	NA	NA	NA	NA	NA	NA	[4]
	**Cerro prieto (USA)**	NA	NA	NA	NA	NA	NA	NA	[1]
	**Salton Sea (USA)**	NA	NA	NA	NA	NA	NA	NA	[5]
	**Rincon (Argentina)**	NA	NA	NA	NA	NA	NA	NA	[1]
	**Soultz-sous-Forêts**	NA	NA	NA	NA	NA	NA	NA	[6]

	**(France)**								
	**Buhl geothermal well (Germany)**	NA	NA	NA	NA	NA	NA	NA	6
	**Landau geothermal well (Germany)**	NA	NA	NA	NA	NA	NA	NA	6
	**Dieng geothermal power plant (Indonesia)**	NA	NA	NA	NA	NA	NA	NA	7
	**Coipasa (Bolivia)**	NA	NA	NA	NA	NA	NA	NA	8
	**Cauchari (Argentina)**	NA	NA	NA	NA	NA	NA	NA	8
**Current study**	**Cornwall (United Kingdom)**	2	2.5	3	29	2.5	141.9	35	**This study**

*NA represents not assigned

Table S9. Comparative study reporting different methods to treat saline water samples for metal removal and Li recovery.

Sr No.	Water samples	Method used for Treatment	pH	Time of treatment (Hrs)	Calcium removal efficiency (%)	Magnesium removal efficiency (%)
**1**	**Artificial seawater cementation solution**	MICP by *Sporosarcina pasteurii*	8.5	24	90	NA
**2**	**Hypersaline produced water**	MICP by ureolytic bacterial consortium	9	240	96	35
**3**	**Brine from Hombre Muerto, Argentina**	Ion Pumping	NA	NA	NA	NA
**4**	**Brine, from north of Argentina**	2 step electro membrane process	13.1	500	99.8	97
**5**	**Brine, from Geothermal power plant, Tibet**	Ion exchange resin	12	1	5	NA
**6**	**Geothermal water, Tibet**	Ion exchange resin	12	12	NA	NA
**7**	**Salt Lake brine from West Taijinar**	crystallization-precipitation method	6.7	3	NA	99
**8**	**Dead sea evaporated end brine**	Chemical precipitation	6.9	NA	NA	NA
**9**	**Arizaro Salt Lake brine**	Electrochemical combined woth precipitation	NA	NA	NA	NA
**10**	**This study**	MICP by *Sporosarcina pasteurii*	9.4	0.15	96	46

Sr No.	Water samples	Manganese removal efficiency (%)	Strontium removal efficiency (%)	% Li recovery	References
**1**	**Artificial seawater**	NA	NA	NA	[9]

	**cementation solution**				
**2**	**Hypersaline produced water**	92.2	94.2	NA	[10]
**3**	**Brine from Hombre Muerto, Argentina**	NA	NA	4.9	[11]
**4**	**Brine, from north of Argentina**	NA	NA	99.8	[12]
**5**	**Brine, from Geothermal power plant, Tibet**	NA	NA	88.68	[13]
**6**	**Geothermal water, Tibet**	NA	NA	88.42	[14]
**7**	**Salt Lake brine from West Taijinar**	NA	NA	93.2	[15]
**8**	**Dead sea evaporated end brine**	NA	NA	40	[16]
**9**	**Arizaro Salt Lake brine**	NA	NA	74.9	[17]
**10**	**This study**	88	91	96	**This study**

*NA represents not assigned

Figure S1. Microbial induced carbonate precipitation in natural brine inoculated (at O.D. 0.1) with *S. pasteurii* (red circles) and *B. subtilis* (black squares). Plots show concentration of Ca (a), Li (b), Mg (c), Mn (d) and Sr (e) in the solution over time. Error bars are the standard error of the mean, n = 3.

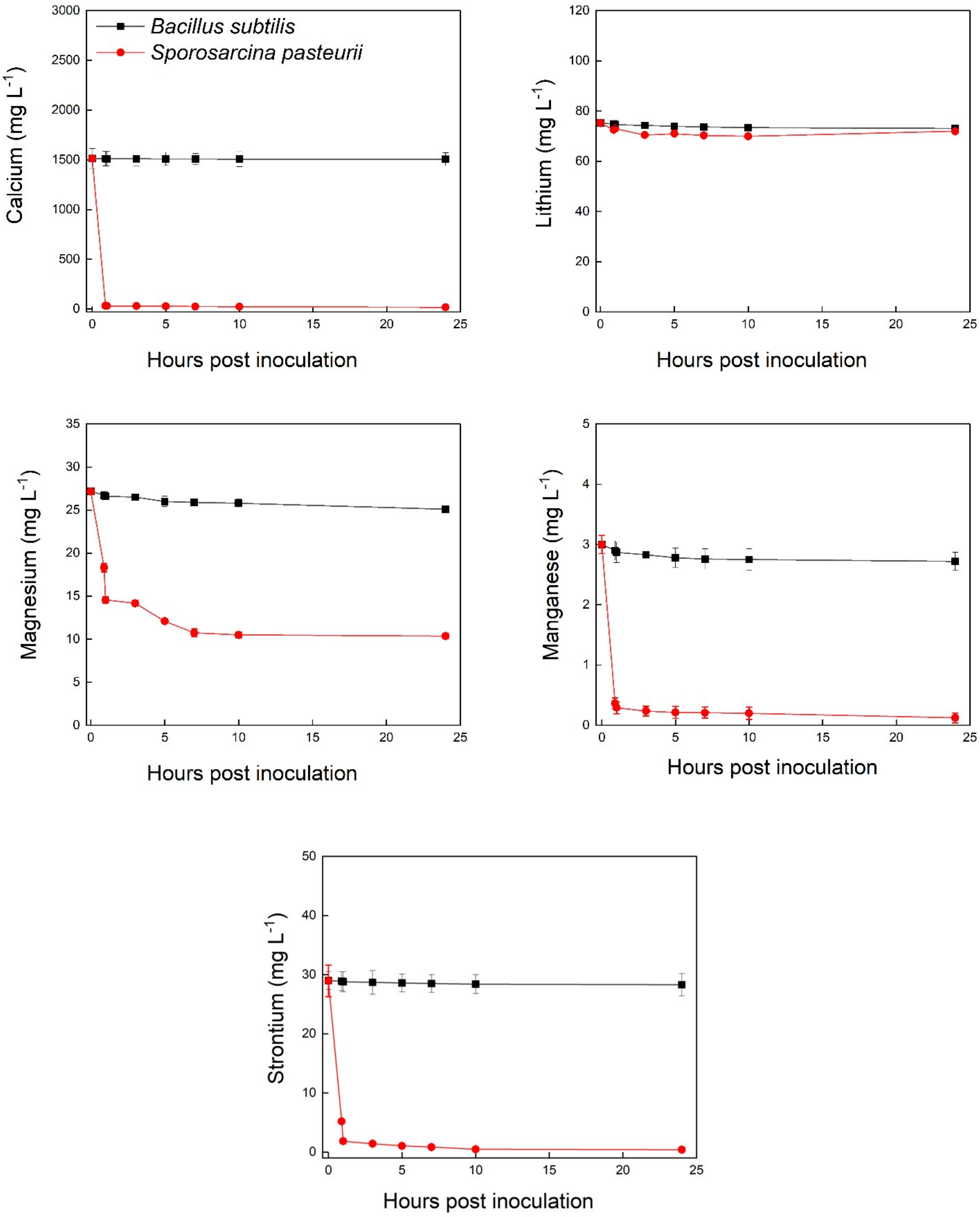

(e)

Figure S2. Microbial induced carbonate precipitation in natural brine inoculated (at O.D. 0.2) with *S. pasteurii* (red circles) and *B. subtilis* (black squares). Plots show concentration of Ca (a), Li (b), Mg (c), Mn (d) and Sr (e) in the solution over time. Error bars are the standard error of the mean, n = 3.

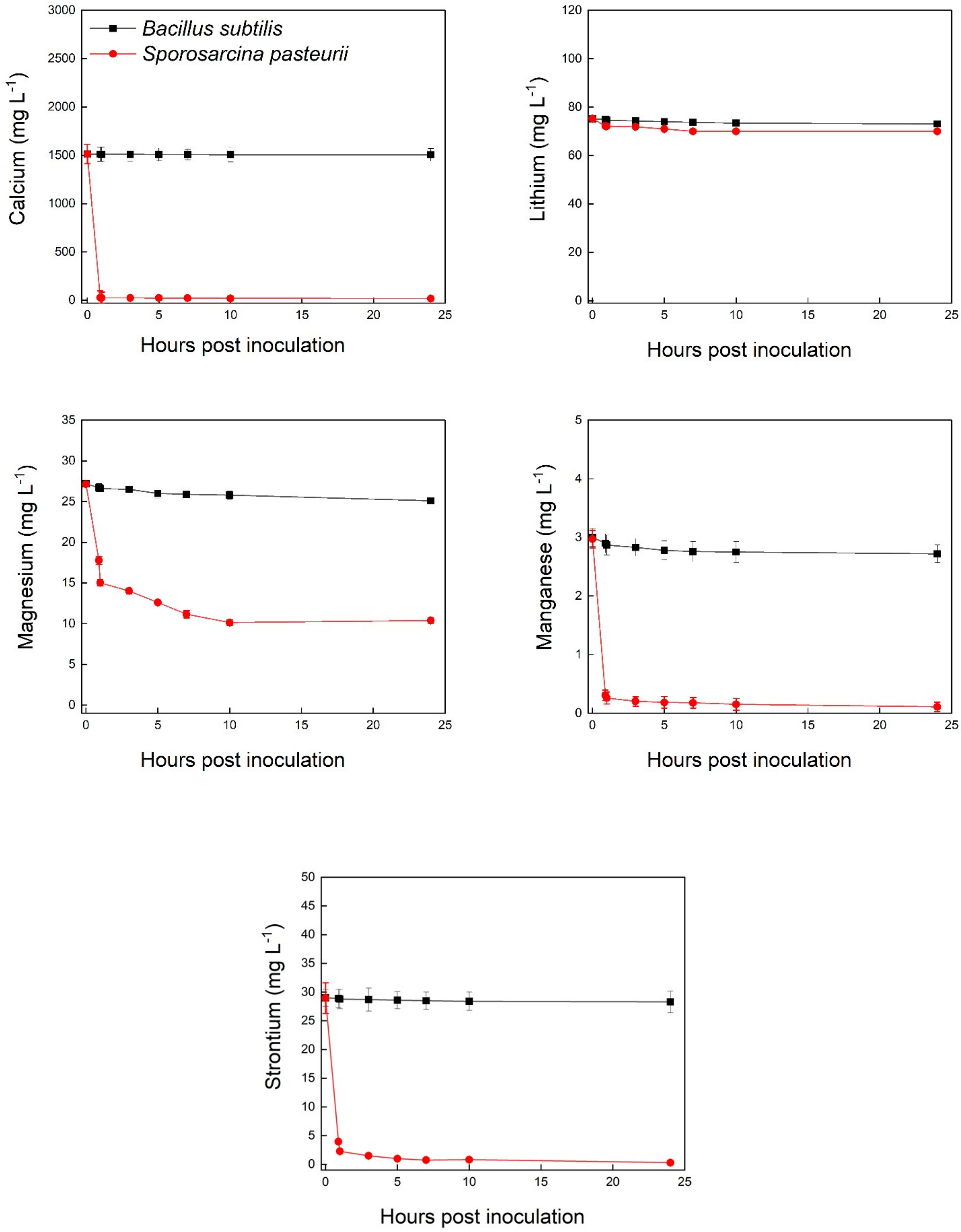

(e)

Figure S3. Microbial induced carbonate precipitation in natural brine inoculated (at O.D. 0.3) with *S. pasteurii* (red circles) and *B. subtilis* (black squares). Plots show concentration of Ca (a), Li (b), Mg (c), Mn (d) and Sr (e) in the solution over time. Error bars are the standard error of the mean, n = 3.

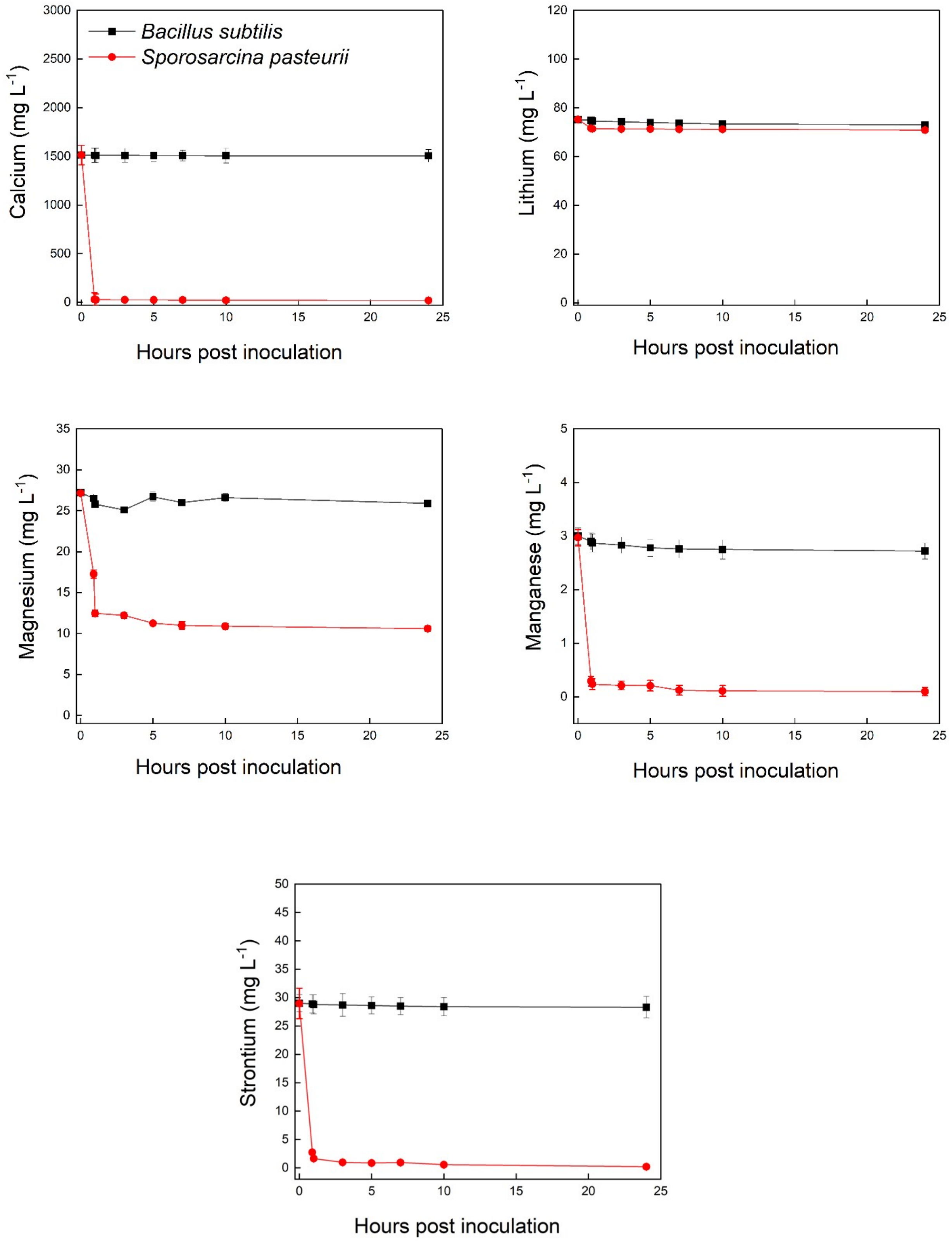

(e)

Figure S4. Microbial induced carbonate precipitation in natural brine inoculated (at O.D. 0.4) with *S. pasteurii* (red circles) and *B. subtilis* (black squares). Plots show concentration of Ca (a), Li (b), Mg (c), Mn (d) and Sr (e) in the solution over time. Error bars are the standard error of the mean, n = 3.

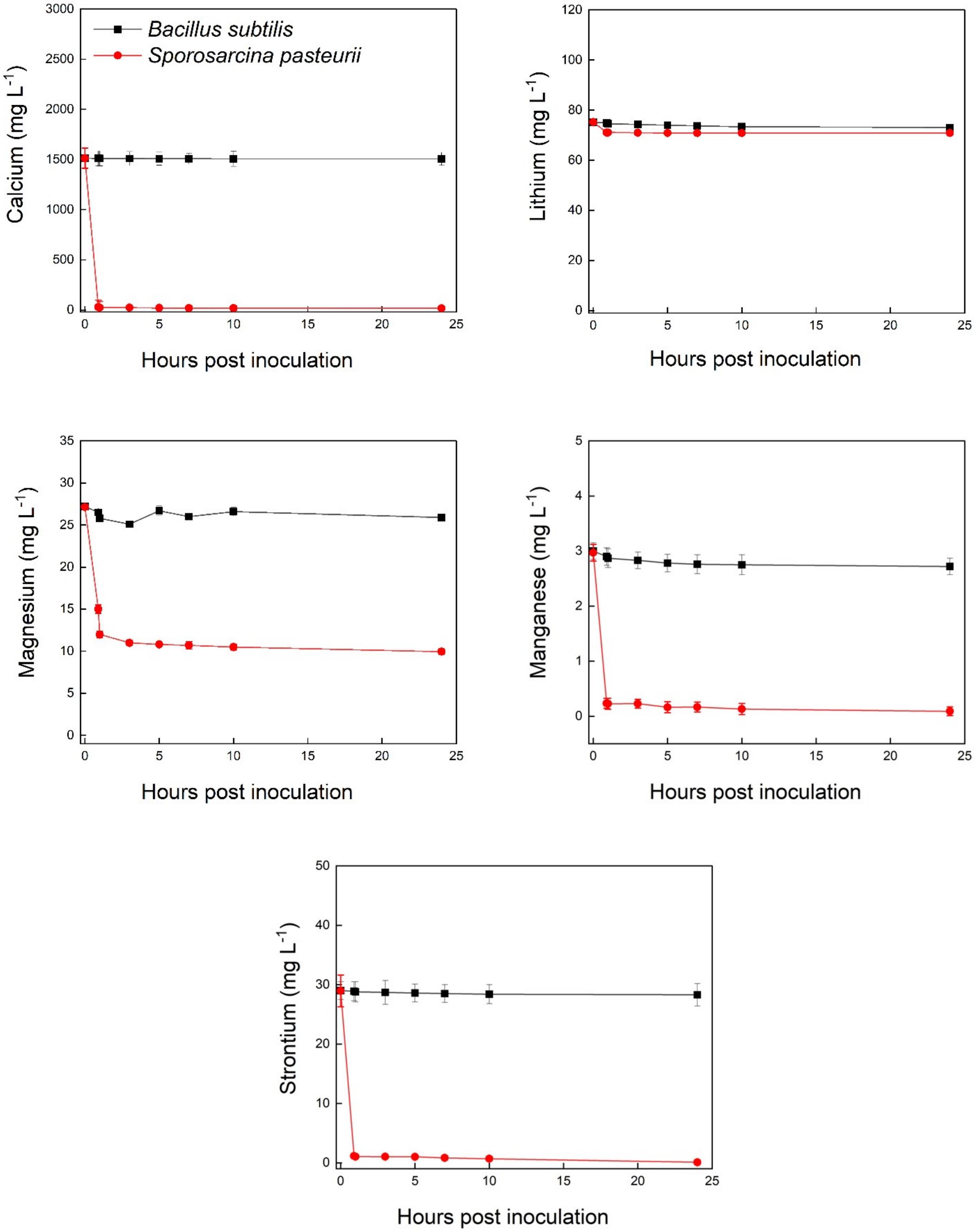

Fig S5 Microbial induced carbonate precipitation in natural brine inoculated with *S. pasteurii* (red circles) and no cell control with only urea added in natural brine (black squares). Plots show concentration of Ca (a), Mg (b), Li (c), Mn (d) Sr (e) and measured pH (f) in the solution over time. Error bars are the standard error of the mean, n = 3.

(e)

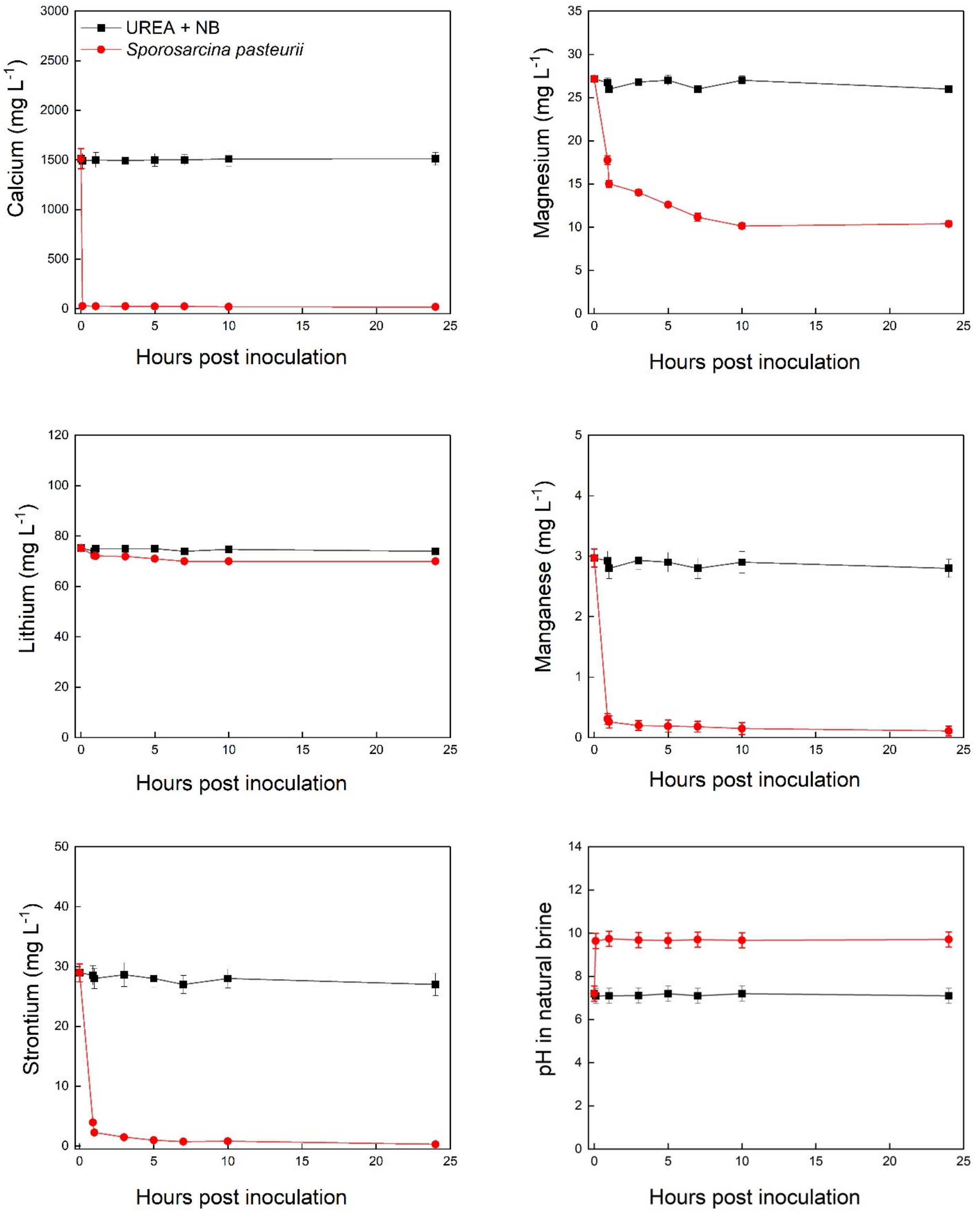
UREA + NB
Sporosarcina pasteurii
Calcium (mg L-1)
Magnesium (mg L-1)
Lithium (mg L-1)
Manganese (mg L-1)
Strontium (mg L-1)
pH in natural brine
Hours post inoculation

Figure S6. Growth performance of *S. pasteurii* (red circle) and *B. subtilis* (black square) in TSB for MICP treatment. Plot show optical density measured at 600 nm in TSB medium (a), pH of TSB medium (b). Error bars are the standard error of the mean, n = 3.

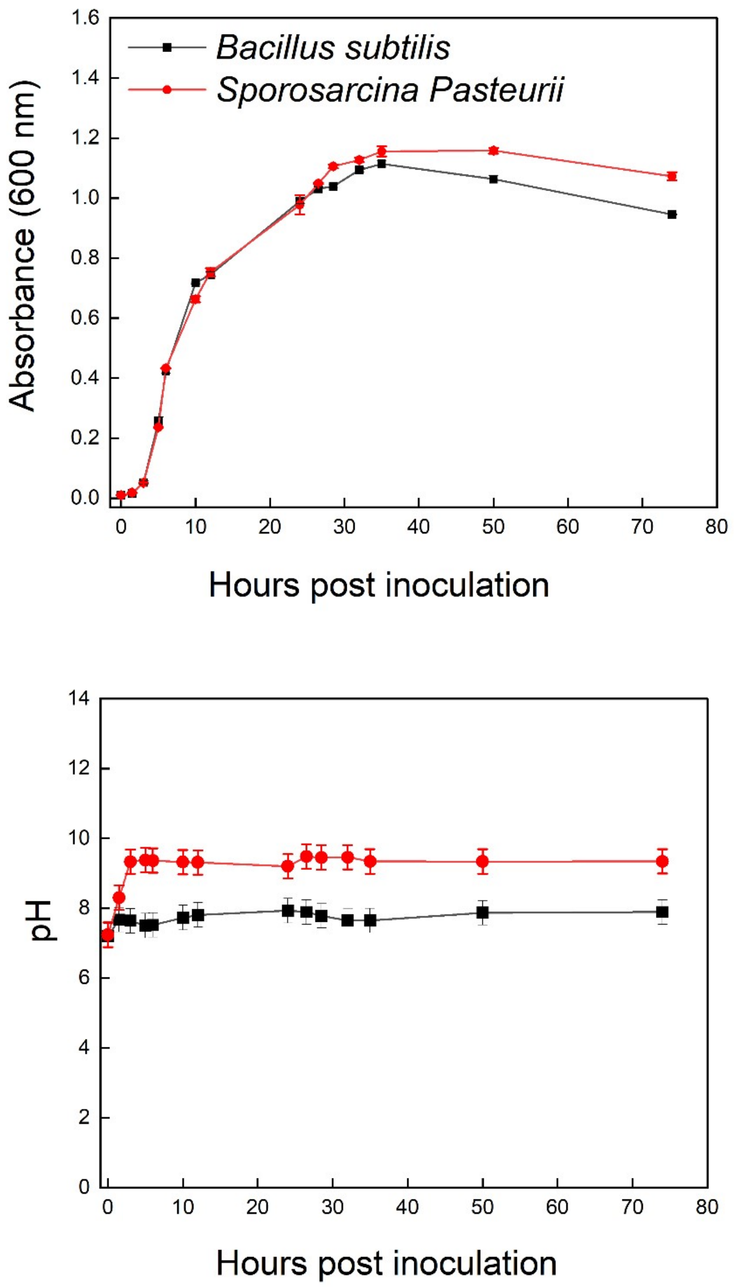

Figure S7. XRD analysis of powdered precipitates with *S. pasteurii* cells in the natural brine solution.

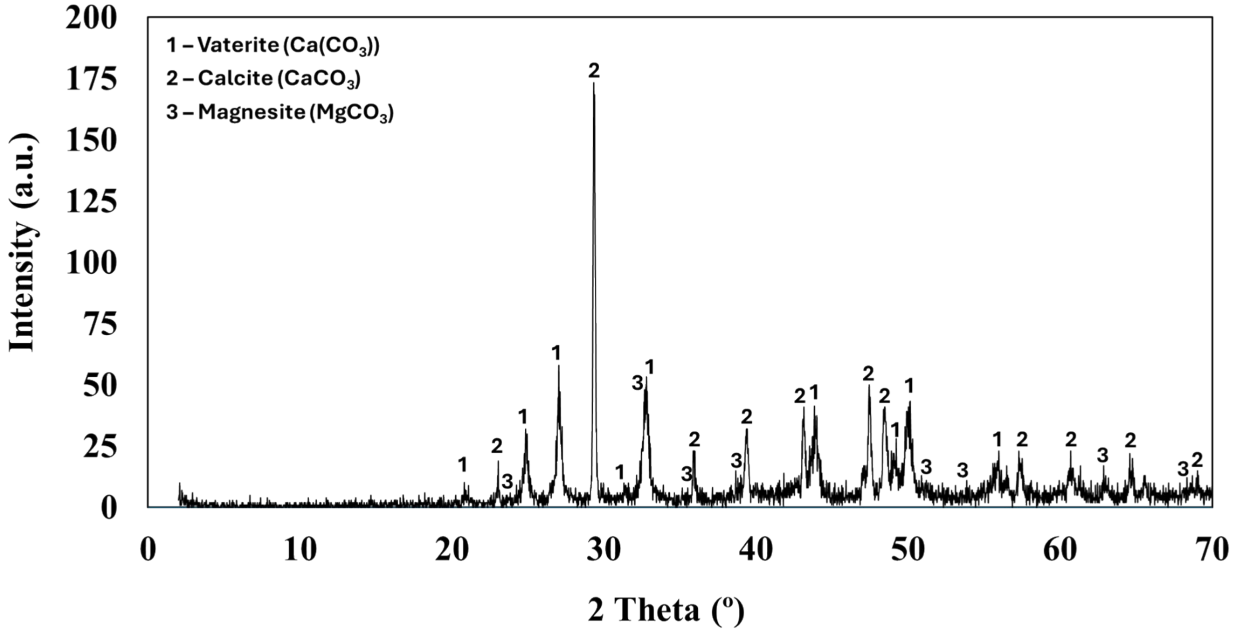

Figure S8. Surface charge on *S. pasteurii* (red circle) and *B. subtilis* (black square). Plot shows zeta potential (mV) in TSB medium at different pH values (2-11). Error bars are the standard error of the mean, n = 3.

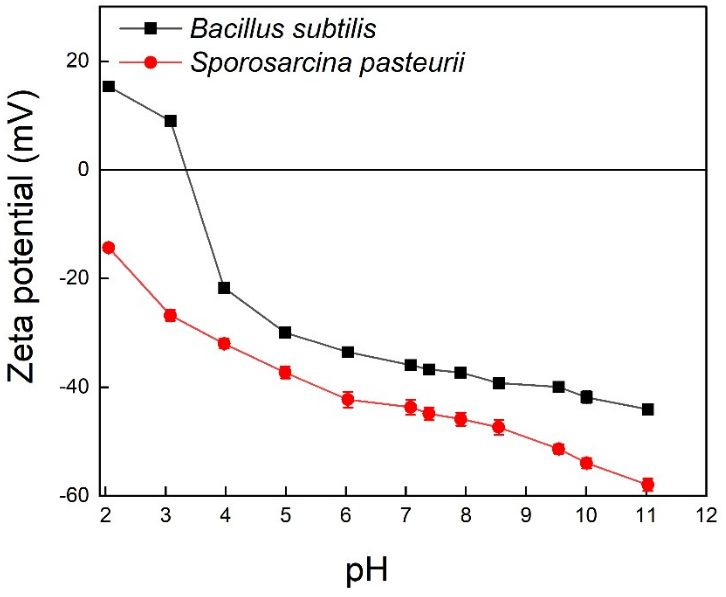

Figure S9. Urea uptake by *S. pasteurii* cultivated using commercial urea (black square) and cow urine-based urea (red circle) in $CaCl_2$ solution. Plot shows urea concentration in $CaCl_2$ solution over time. Error bars are the standard error of the mean, n = 3.

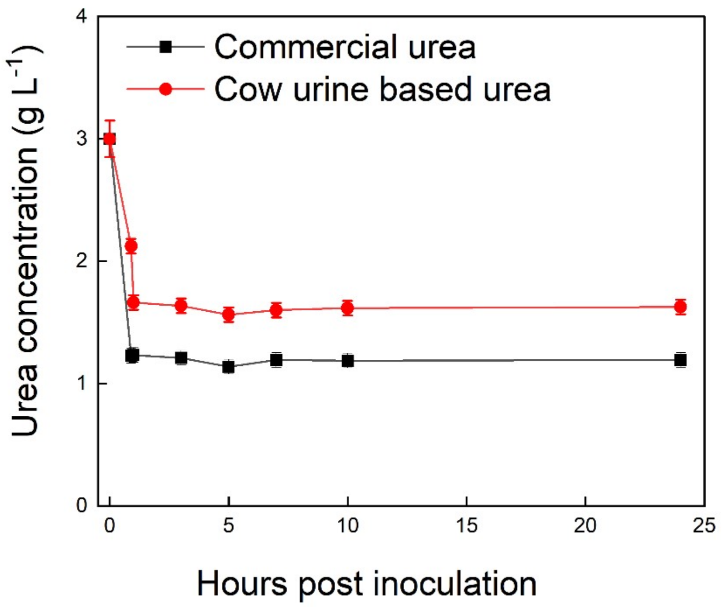

Supplementary References

THIS REFERENCE NUMBERS ARE NOT THE SAME AS IN THE MAIN MANUSCRIPT

1 D. E. Garrett, *Handbook of Lithium and Natural Calcium Chloride*, 2004, 1–476.
2 M. G. Franco, Y. J. Peralta Arnold, C. D. Santamans, R. L. López Steinmetz, F. Tassi, S. Venturi, C. B. Jofré, P. J. Caffe and F. E. Córdoba, *Journal of South American Earth Sciences*, 2020, **103**, 102742.
3 S. Xu, J. Song, Q. Bi, Q. Chen, W.-M. Zhang, Z. Qian, L. Zhang, S. Xu, N. Tang and T. He, *Journal of Membrane Science*, 2021, **635**, 119441.
4 A. Somrani, A. H. Hamzaoui and M. Pontie, *Desalination*, 2013, **317**, 184–192.
5 W. T. Stringfellow and P. F. Dobson, *Energies*, DOI:10.3390/en14206805.
6 B. Sanjuan, R. Millot, Ch. Innocent, Ch. Dezayes, J. Scheiber and M. Brach, *Chemical Geology*, 2016, **428**, 27–47.
7 F. A. Setiawan, E. Rahayuningsih, H. T. B. M. Petrus, M. I. Nurpratama and I. Perdana, *Geothermal Energy*, 2019, **7**, 22.
8 C. F. Baspineiro, J. Franco and V. Flexer, *Science of The Total Environment*, 2020, **720**, 137523.
9 J. Yang and Y. Wang, *Efficiency and Characteristics of MICP in Environments with Elevated Salinity, Diminished Oxygen, and Lowered Temperature: A Microfluidics Investigation*, 2024.
10 L. Hu, H. Wang, P. Xu and Y. Zhang, *Water Research*, 2021, **190**, 116753.
11 V. C. E. Romero, K. Llano and E. J. Calvo, *Electrochemistry Communications*, 2021, **125**, 106980.
12 C. H. Díaz Nieto, N. A. Palacios, K. Verbeeck, A. Prévoteau, K. Rabaey and V. Flexer, *Water Research*, 2019, **154**, 117–124.
13 K. Zhao, B. Tong, X. Yu, Y. Guo, Y. Xie and T. Deng, *Chemical Engineering Journal*, 2022, **430**, 131423.
14 W. Ding, J. Zhang, Y. Liu, Y. Guo, T. Deng and X. Yu, *Chemical Engineering Journal*, 2021, **426**, 131689.
15 X. Lai, P. Xiong and H. Zhong, *Hydrometallurgy*, 2020, **192**, 105252.
16 A. Alsabbagh, S. Aljarrah and M. Almahasneh, *Minerals Engineering*, 2021, **170**, 107038.
17 D. Liu, Z. Zhao, W. Xu, J. Xiong and L. He, *Desalination*, 2021, **519**, 115302.
